# Feasibility and safety of intrathecal transplantation of autologous bone marrow mesenchymal stem cells in horses

**DOI:** 10.1186/s12917-015-0361-5

**Published:** 2015-03-15

**Authors:** Leandro Maia, Fernanda da Cruz Landim- Alvarenga, Marilda Onghero Taffarel, Carolina Nogueira de Moraes, Gisele Fabrino Machado, Guilherme Dias Melo, Rogério Martins Amorim

**Affiliations:** Department of Animal Reproduction, São Paulo State University, District of Rubião Júnior, n/n, CEP: 18618970, Botucatu, São Paulo Brazil; Department of Veterinary Medicine, Maringá State University, Av. Colombo, 5.790, CEP: 87020-900, Maringá, Paraná Brazil; Department of Clinic, Surgery and Animal Reproduction, São Paulo State University, Clóvis Pestano, 793, CEP: 16050-680, Araçatuba, São Paulo Brazil; Department of Veterinary Clinics, São Paulo State University, District of Rubião Júnior, n/n, CEP: 18618970, Botucatu, São Paulo Brazil

**Keywords:** Mesenchymal stem cells, Transplantation, Horse, Matrix metalloproteinases, Neurology

## Abstract

**Background:**

Recent studies have demonstrated numerous biological properties of mesenchymal stem cells and their potential application in treating complex diseases or injuries to tissues that have difficulty regenerating, such as those affecting the central and peripheral nervous system. Thus, therapies that use mesenchymal stem cells are promising because of their high capacity for self-regeneration, their low immunogenicity, and their paracrine, anti-inflammatory, immunomodulatory, anti-apoptotic and neuroprotective effects. In this context, the purpose of this study was to evaluate the feasibility and safety of intrathecal transplantation of bone marrow-derived mesenchymal stem cells in horses, for future application in the treatment of neurological diseases.

**Results:**

During the neurological evaluations, no clinical signs were observed that were related to brain and/or spinal cord injury of the animals from the control group or the treated group. The hematological and cerebrospinal fluid results from day 1 and day 6 showed no significant differences (P > 0.05) between the treated group and the control group. Additionally, analysis of the expression of matrix metalloproteinase (MMP) -2 and −9 in the cerebrospinal fluid revealed only the presence of pro-MMP-2 (latent), with no significant difference (P > 0.05) between the studied groups.

**Conclusions:**

The results of the present study support the hypothesis of the feasibility and safety of intrathecal transplantation of autologous bone marrow-derived mesenchymal stem cells, indicating that it is a promising pathway for cell delivery for the treatment of neurological disorders in horses.

## Background

Recent studies have demonstrated numerous biological properties of mesenchymal stem cells (MSCs), as well their potential application to treat complex diseases or injuries to tissues that have difficulty regenerating, such as those that affect the central and peripheral nervous system. Thus, stem cell therapy is a promising alternative for the treatment of these diseases because of the high capacity for self-regeneration, the low immunogenicity, and the paracrine, anti-inflammatory, immunomodulatory, anti-apoptotic and neuroprotective effects of MSCs.

During ischemic damage or severe tissue damage, MSCs can be attracted to the site of injury, where they secrete bioactive factors that influence the process of tissue repair and regeneration [1]. According to Baraniak and McDevitt [2], stem and progenitor cells are able to produce and secrete a large number of factors, including growth, angiogenic, antifibrotic, anti-inflammatory and immunosuppressive factors and compounds responsible for the homeostasis of the extracellular matrix (collagen, TIMPs and matrix metalloproteinases (MMPs), as well as antioxidants and anti-apoptotic molecules that play important roles in the regenerative process. In addition to producing cytokines, chemokines, growth factors and extracellular matrix molecules, it is noteworthy that stem/progenitor cells play an important role in the consume of pro-apoptotic factors and inflammatory molecules [2]. All these properties are great attractions for the use of cell therapy in many diseases that affect animals and humans. However, preclinical and clinical trial data are fundamental to provide the scientific basis in support of stem cell therapy. The therapeutic window, the strategy of cell delivery, the cell doses and the possible secondary effects should be evaluated in clinical studies [1]. Because horses are commonly affected by musculoskeletal, neurological and reproductive disorders, the equine species can be used as a model for experimental MSCs therapy.

MSCs derived from the bone marrow (BM) and adipose tissue are the two most common types of stem cells used in therapeutic approaches to repair and/or regenerate tissues in horses [3]. The studies involving MSCs therapy of horses have focused mainly on osteoarticular and tendon injuries [4-9], with few reports of its use in other tissues, such as the nervous system. Thus, the application pathways most used until now for the clinical treatment of horses are intralesional and intra-articular. Preclinical studies that evaluated the safety of MSCs transplantation by other pathways, such as intrathecal, are lacking in equine medicine. An intrathecal injection is less invasive than an intralesional injection, can extensively deliver cells through the cerebrospinal fluid (CSF) [10] and is a more feasible routine for treatment of equine CNS injuries.

In this context, the purpose of this study was to evaluate the feasibility and safety of intrathecal transplantation of autologous bone marrow mesenchymal stem cells (BM-MSCs) in horses.

## Results

### Cultivation, characterization and differentiation capacities of the BM-MSCs

The cells from the mononuclear fraction of the BM exhibited adherence to the culture dish between 24 and 48 hours and fibroblastoid morphology starting at four days of cultivation. Confluence (≥80%) and homogeneity of the culture were obtained after approximately three week of cell culture.

The immunophenotyping analysis revealed a high level of expression of the CD90 marker $\left(\overline{x}=97\pm 1.3\%\right)$, a lower level of expression of the CD44 marker $\left(\overline{x}=71\pm 10.7\%\right)$ and the lack of expression of the CD34 marker $\left(\overline{x}=0.8\pm 0.2\%\right)$.

The differentiation potential of the MSCs was demonstrated *in vitro* by a positive response to the osteogenic, adipogenic and chondrogenic differentiation media by the tenth, eighth and twenty-first day of exposure, respectively.

**Figure 1:**
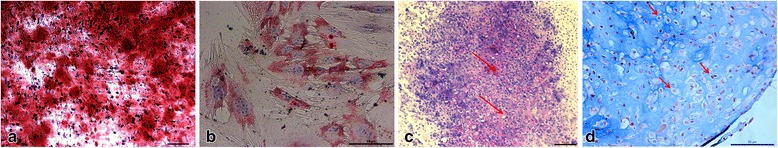
**Differentiation assay of mesenchymal stem cells for osteogenic, adipogenic and chondrogenic lineages. (a)** Osteogenic differentiation. Note calcium deposits stained with Alizarin red. **(b)** Adipogenic differentiation. Note the intracellular presence of lipid droplets stained with Oil red. **(c, d)** Chondrogenic differentiation. **(c)** Toluidin blue staining. Note the presence of metachromatic areas in pink (red arrow) indicating the presence of extracellular matrix containing proteoglycans. **(d)** Alcian blue staining. Note the presence of areas in blue indicating the presence of extracellular matrix containing proteoglycans beyond the presence of gaps (red arrow) possibly containing chondrocytes. Bar = 50 μm.

The MSCs that initially had a fibroblastoid morphology acquired a predominantly polygonal morphology after exposure to the osteogenic medium and deposited large amounts of calcium-rich extracellular matrix, as shown by positive staining with Alizarin red (Figure 1a).

Adipogenic differentiation was confirmed by the deposition of lipid droplets in the cytoplasm, as demonstrated by positive staining with oil red O (Figure 1b). Chondrogenic differentiation was confirmed by the deposition of a hyaline matrix rich in proteoglycans, as demonstrated by positive staining with toluidine blue and Alcian blue (Figures 1c and d).

### BM-MSCs transplantation

The anesthetic protocol used for the BM-MSC transplantation, including the CSF tap, proved to be appropriate and safe for both groups. No animal showed clinical alterations or allergic reactions that could be attributed to the BM-MSC transplantation, compared with the control group (CG).

### Clinical and neurological evaluations

Neither clinical nor neurological alterations were observed in the treated group (TG) or CG animals during the exams conducted pre- and post-transplantation. All the animals exhibited a normal locomotion pattern (grade 0), and neurological deficits were not detected, according to the classification of Mayhew *et al.* [11]. In addition, no significant alterations in the integrity of the brain (mental state, behavior, head position and cranial nerve functions) were observed during the neurological examinations performed for both groups.

### Hematological analysis

The median values obtained for the hematological variables (hematocrit, total protein, platelets, fibrinogen, total leukocytes, neutrophils, lymphocytes, basophils and monocytes) before (Day 1) and after transplantation (Day 6) are presented in Table 1. There were no significant differences (P > 0.05) between the values for the TG and CG groups or between the values at the chosen time points. However, the only variable that increased (p <0.05) in both groups pre and post-transplantation was basophil, but there was no difference between groups. It is noteworthy that this increase was not accompanied by eosinophilia.

**Table 1:** **Median of hematological variables before and after the treatments of treated groups (TG) and control (CG)**

**Variables**	**Before transplantation**		**After transplantation**	
	**Groups**		**Groups**	
	**CG**	**TG**	**CG**	**TG**
**Hematocrit (%)**	36	33	29	32
**Protein (mg/dL)**	7.2	7.6	7.0	7.6
**Platelets (plt/** ***μL*** ^***−1***^ ***)***	182000	151000	193000	176000
**Fibrinogen (mg/dL)**	200	200	400	400
**Leukocytes** ***(cells μL*** ^***−1***^ ***)***	7300	11000	7300	8700
**Neutrophils**	3790	4290	4200	5307
***(cells μL*** ^***−1***^ ***)***				
**Lymphocytes**	2714	4712	2709	3132
***(cells μL*** ^***−1***^ ***)***				
**Eosinophils**	448	284	248	174
***(cells μL*** ^***−1***^ ***)***				
**Basophils**	0	0	126*	315*
***(cells μL*** ^***−1***^ ***)***				
**Monocytes** ***(cells μL*** ^***−1***^ ***)***	192	426	248	312

*Asterisk represents difference (P < 0.05) between moments (before and after transplantation) by the Wilcoxon test.

All values were presented as median.

### CSF analysis

The CSF samples collected from the animals in both groups were clear, colorless and not coagulated, both pre- and post-transplantation.

No significant differences (p > 0.05) between the CG and the TG pre- (day 1) and post-transplantation (day 6) were observed in the CSF density or pH, or the contents of red blood cells, nucleated cells, globulins, proteins, glucose, and pro-MMP-2, as shown in Table 2. Moreover, no significant changes (p > 0.05) in the CSF data obtained pre- and post-BM-MSC transplantation were observed in the TG or the CG.

**Table 2:** **Median of CFS variables before and after the treatments of treated groups (TG) and control (CG)**

**Variables**	**Before transplantation**		**After transplantation**	
	**Groups**		**Groups**	
	**CG**	**TG**	**CG**	**TG**
Density	1006	1006	1006	1006
pH	8.5	8.5	8.5	8.5
Red blood cells	0	1	3	1
(cells μL^−1^)				
Nucleated cells (cells μL^−1^)	0	0	4	0
Globulins	0	0	0	0
Proteins (m*g dL* ^*−1*^)	34	46.1	42.5	58.2
Glucose (m*g dL* ^*−1*^)	54	57	57	55
pro-MMP2 (AU)	34.43	31.86	42.8	28.69

Differences (P < 0.05) between moments and treatments were not observed for none of the variables.

AU: arbitrary units.

All values were presented as median.

### Expression of MMP-2 and -9 in CSF

Only the presence of pro-MMP-2 (inactive/latent) was detected in the CSF from the animals in the two groups; the differences between the values for the groups and the values for the same group at the different time points were not significant (P > 0.05) (Table 2). Latent or activated forms of MMP-9 were not detected in any of the CSF samples.

## Discussion

In the present study, the safety of MSCs transplantation through the CSF was demonstrated by the results obtained from laboratory analysis and by the absence of alterations in the studied groups. The most interesting finding of our study was the absence of the expression of MMP-2 and MMP-9 in the CSF, particularly in their activated forms, after BM-MSC transplantation.

### Characterization and differentiation assays of the MSCs

In the present study, the BM-MSCs isolated from the horses were characterized according to the criteria established by the International Society of Cellular Therapy for human MSCs [12]; i.e., they demonstrated the characteristics of adherence to plastic, fibroblastoid morphology, high expression of the CD90 marker (>95% positive), no expression of the hematopoietic cell marker CD34, and they responded positively to osteogenic, adipogenic and chondrogenic differentiation media. Furthermore, the expression of CD44 (hyaluronic acid-receptor), a surface glycoprotein important for the interactions and cell adhesion of MSCs, was also observed.

Some of these characteristics of the MSCs observed by our group have also been reported in studies that characterized the MSCs from equine adipose tissue [8,13], bone marrow [14-16], peripheral blood [17], the matrix of the umbilical cord [18], amniotic fluid [19], gingival tissue and periodontal ligaments [20].

### BM-MSC transplantation

Concern with the safety of BM-MSCs intrathecal transplantation through the atlanto-occipital space of the horses in the TG guided the selection of the vehicle to be for the cells and the method of preparing the cells. Thus, the BM-MSCs were washed three times to remove residual fetal bovine serum (FBS) that could induce an immune response in the animals. This measure appeared to be adequate because the animals displayed no changes after the transplantation. There is strong concern regarding the use of FBS in the culture medium, particularly for humans, considering that in addition to the possibility of immune reactions, prions, viruses and zoonotic agents could be transferred [21,22]. According to Dimarakis and Levicar [21], serum contains a variety of proteins that can bind to the cultured cells, thereby serving as antigens for immunological reactions after transplantation. Due to the controversy regarding the use of FBS, Toupadaskis *et al.* [23] used equine MSCs to study the possibility of substituting FBS with autologous serum (AS), and showed that the rate of cell proliferation with FBS was higher (P <0.05) than with AS. Therefore, the authors suggested that substituting only AS may not be a viable alternative for cell expansion under the current experimental conditions and that further studies must be directed at determining methods whereby immunogenicity can be reduced without affecting the growth rate of the MSCs. Additionally, these methods could include the initial culture and cell expansion with FBS and subsequent cell expansion with AS to reduce the immunogenic potential [23].

The phosphate-buffered solution (PBS) vehicle used for transplantation/in both groups was shown to be adequate and safe, as demonstrated by the absence of complications, particularly in the CG. This vehicle has been used in experimentally induced tendinopathy in horses [7].

The concentration of the BM-MSCs transplanted in this pre-clinical study was similar to that used by Lim *et al.* [24] for lumbar administration to a rat model of stroke induced by occlusion of the middle cerebral artery. In their study, doses of 10^5^ and 10^6^ MSCs derived from the blood of human umbilical cords that were administered intrathecally had significant effects on recovery from ischemic damage. These findings support the hypothesis that BM-MSC transplantation via an intrathecal pathway is feasible and safe, and suggest great prospects for the use of cell therapy in treating neurological diseases in other species.

### Clinical evaluation

The absence of clinical alterations, including neurological signs, observed in the TG under the conditions of this study demonstrated that the procedures performed did not dramatically change the neural environment, indicating that a CSF pathway can be used for BM-MSCs transplantation in horses. In laboratory studies of animals and humans, the CSF pathway has been used for the transplantation of mononuclear cells and allogeneic MSCs, without evidence of serious adverse effects [24,25].

In a study conducted with 114 humans affected by degenerative conditions (paraplegia, ataxia and multiple sclerosis), Yang *et al.* [25] performed a total of 592 intrathecal and intravenous administrations of allogeneic mononuclear cells from umbilical cord blood without observing serious adverse effects. The most common collateral effect (19/592, 3.2%) was headache, which was attributed to postural hypotension, a known complication of lumbar puncture that resolves spontaneously without the need for drastic interventions.

### Hematological and CSF analyses

The CSF performs four major functions in the central nervous system (CNS), including the physical support of the neural structure, excretion, intracerebral transport and control of the chemical environment of the CNS [26]. Among these functions, the one that motivated us to conduct this study was the possibility of the CSF transporting MSCs, which have the potential to migrate, to sites of injury.

The transplantations performed in our study did not cause significant differences in the CSF values (p > 0.05) of the TG and CG. The density of the CSF [27] and its contents of erythrocytes, nucleated cells, protein and glucose [28] pre- and post-transplantation were within the normal ranges for the equine species. The pre- and post-transplantation pH values obtained using reagent strips were also considered normal in the laboratory where the analysis was conducted.

The median pre- and post-transplantation values for the pro-MMP-2 contents of the CSF from the CG and TG were similar to the average value (43.12 ± 13.90) determined by Melo *et al.* [29] in healthy dogs that were used as controls in a study conducted recently to evaluate the expression of MMP-2 and MMP-9 in the CSF and serum of dogs with neurological signs and visceral leishmaniasis. Our results cannot be compared with those of other studies conducted with equine species due to the difficulty of finding information in the literature about the MMP contents in the CSF of this species. Thus, the values obtained, particularly the pre-transplantation values for pro-MMP-2, will serve as a reference for further studies.

Similarly, the values for the hematological variables also did not differ (P > 0.05) between the groups or between the time points studied, demonstrating that the treatments did not affect those variables.

### Expression of MMP-2 and -9 in CSF

MMPs are a family of zinc- and calcium-dependent endopeptidases that are responsible for degrading and remodeling the extracellular matrix, including its collagen, elastin, gelatin, proteoglycan and glycoprotein components [30]. An important characteristic of MMPs is their latency. These proteases are secreted in a pro-form (inactive/latent), requiring activation by a variety of mechanisms before becoming functional [31]. In the present study, the activity of only pro-MMP-2 (gelatinase A) was identified in the CSF, with no difference (P > 0,05) between the values of the groups or of each group at the two time points studied. According to Rosenberg [31], gelatinase A is a constitutively expressed molecule that is normally found in brain tissue and CSF. MMP-2 has been demonstrated in the astrocytic processes of normal brain, particularly those adjacent to vessels, the ependymal cells and the pia mater. The presence of MMP-2 in the astrocytic processes near the surface of the brain suggests that this metalloenzyme may play a role in the homeostasis of the brain fluid or in regulating the blood–brain barrier. Bergman *et al.* [32], who studied 23 clinically healthy dogs, also obtained results similar to ours, observing only the presence of pro-MMP-2 and the absence of MMP-9 in the CSF.

The lack of evidence for latent or active MMP-9 (gelatinase B), particularly in the group treated with MSCs, supports the hypothesis that the transplantation pathway tested by our group is safe, considering that according to Rosenberg [31], MMP-9 is markedly dysregulated under the inflammatory conditions of many diseases. High levels of MMP-9 have been observed in the CSF of human patients and animals affected by neurological diseases that involve intense neuroinflammation, such as visceral leishmaniasis [29], traumatic cerebral injury [33], meningitis [34,35] and multiple sclerosis [36].

## Conclusion

Intrathecal transplantation of autologous BM-MSCs in horses does not cause clinical alterations, particularly in the variables evaluated in the neurological examinations and the hematological and CSF analyses, including the expression of MMPs. Therefore, this pathway for the delivery BM-MSCs was shown to be feasible and safe, raising the possibility of performing future clinical trials to treat neurological diseases in horses.

## Methods

### Animals

Ten healthy crossbred horses of both sexes (5 males and 5 females), aged between 4 and 12 years 300 to 500 Kg were used. The animals were selected based on prior clinical, hematological and neurological evaluations.

The experimental protocol (number 76/2009 - CEUA) was approved by the ethics committee of São Paulo State University, Botucatu, Brazil. All the procedures were performed according to the international guidelines for the care and use of experimental animals.

### Experimental delineation

The selected animals were randomly divided into two groups; one group was transplanted with BM-MSC (TG, n = 5) and the other, which was the control group, received PBS (CG, n = 5). First, the bone marrow (BM) obtained using needle aspiration was used for to isolate, expand and characterize the BM-MSCs. After the characterization, the cells were transplanted intrathecally into the TG through the cisterna magna. In this same period, the animals from the CG received the same volume of PBS by the same pathway.

**Figure 2:**
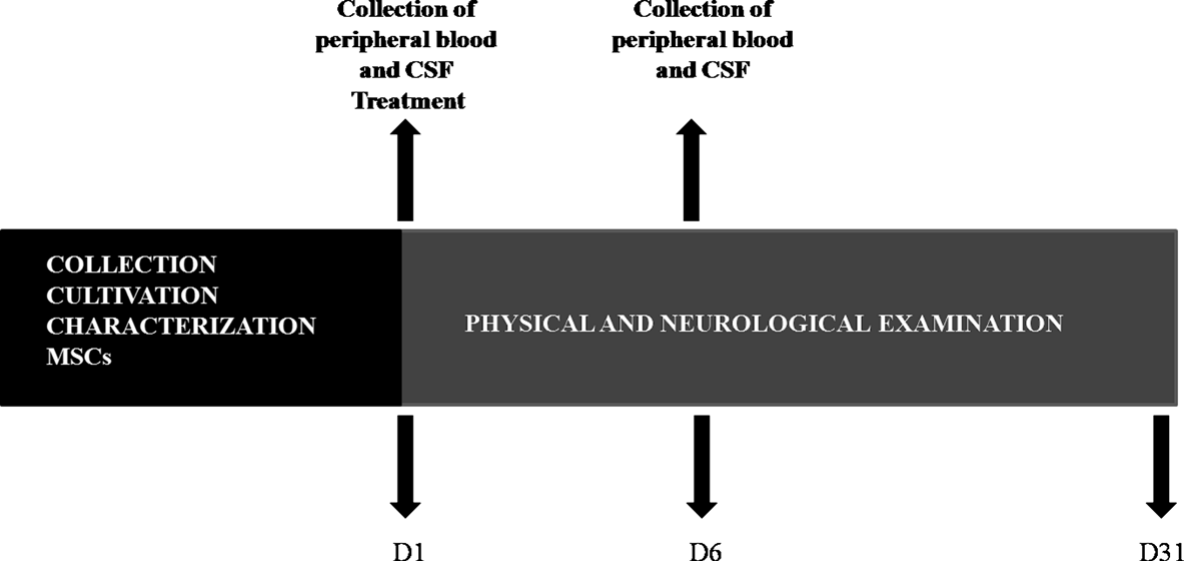
**Timeline.** Prior to the treatment, bone marrow was collected, cultivation and mesenchymal stem cells (MSCs) characterized. On day 1 (D1) and 6 (D6) peripheral blood and CSF samples were obtained. All the animals were monitored by physical and neurological examination for 30 days during the experiment.

The safety of intrathecal BM-MSC transplantation was monitored by daily clinical and neurological examinations during the period of thirty days (day 1 until day 31), as well as by hematological and CSF analysis, including examining the expression of the latent and activated forms MMP-2 and −9 in the CSF (Figure 2).

### Bone marrow collection

BM aspiration was performed according to the methodology described by Barreira *et al.* [37], with modifications. For this, the animals were maintained on quadrupedal position, physically restrained and sedated with 0.5 mg kg^−1^ xylazine (Sedomin®, Koning, ARG). Then it was performed shaving of an area of 5 × 20 cm related to the sternum of each horse. After identification of the fifth sternebrae it was performed antisepsis and local anesthetic block (Xylestesin® 2% Cristália, BRA). Once fixed, the bone marrow needle, Komiyashiki model, within the sternum, the mandrel was removed and proceeded to the aspiration of BM with the aid of two 20 mL syringes containing 2 mL of heparin (Hemofol, Cristália, BRA) and 2 mL of PBS, pH 7.2 (PBS 1×, LCG Biotechnology, BRA). After collection, the samples were identified and forwarded to the laboratory for processing.

### Isolation and cultivation of BM- MSC

The isolation and cultivation of BM-MSCs were performed according to the methodology described by Maia *et al.* [38]. The mononuclear fraction from the samples of BM was cultured in low-glucose DMEM /F12 (1:1), 20% fetal bovine serum, penicillin/streptomycin (1%) and amphotericin B (1.2%) (Gibco Invitrogen, USA) at 37.5°C in a humid atmosphere containing 95% air and 5% CO2. The maintenance medium was changed every 2 or 3 days until a minimum of 80% cellular confluence was reached, and then the cells were recovered by trypsinization for characterization and use in treatment.

### Flow cytometric analysis of cell surface markers

The immunophenotypic analysis of the BM-MSCs was performed on the primary cultured cells, using a FACSCalibur cytometer (Becton Dickinson and Company, USA) using monoclonal mouse anti-rat CD90 (clone OX7, 1:100, Caltag Laboratories, USA) and mouse anti-human CD34 (clone 581/CD34, 1:50, Becton Dickinson and Company, USA) antibodies labeled with fluorescein isothiocyanate (FITC) and a mouse anti-horse CD44 (clone CVS18, 1:100, AbD Serotec, USA) antibody that was detected using an FITC-conjugated goat anti-mouse secondary antibody (1:200, Molecular Probes, USA). During the analysis, 10,000 events were recorded.

### Assays of osteogenic, adipogenic and chondrogenic differentiation

After reaching confluence in the primary culture, BM-MSCs were trypsinized and seeded at a density of 2 × 10^5^ MSCs/well, in six-well plates (Sarstedt, USA). After 48 hours, the maintenance medium was removed and media for osteogenic or adipogenic differentiation StemPro (Gibco Invitrogen, USA) were added to the subcultures, in triplicate, according to the manufacturer’s recommendation with modifications. The adipogenic medium was supplemented with 5% rabbit serum. The media were changed every 2 to 3 days and confirmation of osteogenic and adipogenic differentiation was obtained, respectively, by demonstrating the deposition of a calcium-containing matrix using the histological method of staining with 2% Alizarin red, pH 4.2 and the presence of intracytoplasmic lipid droplets by staining with 0.5% oil red O (Sigma-Aldrich Corp., USA).

For chondrogenic differentiation, the BM-MSCs were cultivated at a density of 10^6^ MSCs/mL in a 3D pellet in a Falcon tube (15 mL) for 21 days in StemPro chondrogenic differentiation medium that was changed every three days. To confirm that chondrogenic differentiation had occurred, the pellets were stained with Alcian Blue, pH 2.5, and toluidine blue, pH 1, which detect proteoglycans [39].

### Collection of blood and hematological analysis

The blood samples for the hematological analyses were collected from the TG and CG animals immediately before (day 1) and on six days (day 6) after the intrathecal transplantation.

Aliquots of 4 mL of blood were collected by puncture of the external jugular vein into tubes containing anticoagulant (Vacutainer BD, USA). The blood in the EDTA-containing tubes were used to determine the hematocrit, total plasma protein, number of platelets, fibrinogen content, total leukocyte count and the differential cell count. The total leukocytes and platelet counts were performed using a cell counter (HemaScreen 18, Ebram, BRA).

### Collection and analysis of CSF

CSF was collected from the animals in both groups through the atlanto-occipital space, according to the technique described by Mayhew [40], immediately before BM-MSC transplantation (TG) or PBS inoculation (CG) (day 1) and subsequently in day 6. All horses were sedated with 0.5 mg/kg xylazine (Sedomin®, Koning, ARG), followed by induction of anaesthesia with ketamine (3 mg/kg) (Cetamin, Rhobifarma, BRA) associate with diazepan (0,1 mg/kg) (Compaz, Cristália, BRA). After orotracheal intubation, anaesthesia was maintained with isoflurane (Isoforine, Cristália, BRA) in oxygen. Ventilation was controlled with a tidal volume of 10 ml/kg and 10 breaths per minute.

The physical variables of the CSF that were evaluated were the appearance, color and coagulation; the density was determined using refractometry and the pH determined using a reagent strip. For the cytological analysis, the total number of cells in the undiluted CSF samples was counted using a Neubauer chamber, and the differential counts were performed after cytocentrifugation (Revan centrifuge 2000 D).

In the biochemical analysis, the concentrations of protein and glucose in the CSF samples were determined using commercial kits (Micropote kit, Doles, BRA and Glucose kit, Katal, BRA, respectively), according to the manufacturers’ recommendations.

The possible presence of globulins in the CSF was determined by the Pandy test, in which 1 mL of Pandy reagent is mixed and homogenized with a few drops of CSF. In the case of a positive test, the results for the intensity of the turbidity observed macroscopically were represented using one to four crosses.

Immediately after the collection, CSF samples from each of the animals in the TG and the CG were cryopreserved for subsequent evaluation of their contents of MMP-2 and MMP-9 using zymography.

### BM-MSC transplantation

Prior to transplantation, the BM-MSCs were trypsinized and washed three times with filtered DMEM medium by centrifugation at 250 g to remove residual fetal bovine serum which could induce allergic and/or immune responses in the animals in the treated group (TG, n = 5). After this procedure, the pelleted BM-MSCs were suspended in 2 mL of PBS for immediate transplantation. The TG received on average approximately 1 × 10^6^ BM-MSCs suspended in 2 mL of PBS intrathecally through the atlanto-occipital space using the technique and anesthesia described previously. The control group (CG, N = 5) received 2 mL of PBS (placebo) by the same pathway.

### Clinical and neurological evaluations

After treating of the CG and TG animals, physical examinations were performed daily for thirty days, to evaluate behavior, posture, degree of hydration, staining of the mucosa, capillary refill time, heart rate, respiratory rate, body temperature and bowel movements and to determine the body condition score.

The evaluation of the integrity of the brain (mental state, behavior, head position and cranial nerve functions) and spinal cord was performed as described by Malikides *et al.* [41] and Mayhew [42].

During the neurological examinations of the TG and CG animals, locomotion was evaluated using the classification system of ataxia and paresis described by Mayhew *et al.* [11], as follows:

- *Degree 0 (normal)* – deficit not detected.
- *Degree 1* – deficit barely detected at a normal gait or posture.
- *Degree 2* – deficit easily detected, and exaggerated by backing, turning, swaying, loin pressure and neck extension.
- *Degree 3* – deficit very prominent at a normal gait with a tendency to buckle or fall with backing, turning, swaying, loin pressure and neck extension.
- *Degree 4* – stumbling, tripping and falling spontaneously at a normal gait, and more severe deficits.

### Analysis of MMPs using the technique of gelatin zymography

The latent (pro-) and activated forms of MMP-2 and −9 were analyzed using zymography and densitometry, according to the methods described by Melo et al. [29] and Marangoni et al. [43]. Briefly, a volume containing an equal amount of total protein was incubated in the sample buffer (125 mM Tris–HCl, pH 6.8; 20% v/v glycerol, 4% w/v SDS, 0.2% w/v bromophenol blue) without boiling and submitted to electrophoresis with polyacrylamide gels (10%) copolymerized with gelatin (G8150-100G, Sigma–Aldrich). The gels were rinsed in 2.5% Triton X-100 for 30 min and incubated in the enzymatic activation buffer (50 mM Tris, 200 mM NaCl, 5 mM CaCl_2_, 0.2% w/v Brij-35, pH 7.5), for 20 h at 37°C with gentle shaking, which allows gelatin digestion by both latent and active forms of MMPs. The gels were stained (0.5% w/v Coomassie brilliant blue R-250, 45% v/v methanol, 10% v/v glacial acetic acid) for 30 min, and destained in the same solution without the dye for 45 min. MMP levels were assessed using gelatinolytic activity, indicated as clear bands against the dark blue background. MMP identity and normalization between gels was achieved with human recombinant MMP-2 (PF037, Calbiochem) and MMP-9 (PF038, Calbiochem). The gels were digitalized and the integrated density of the bands, expressed as arbitrary units, was calculated using the open-access software ImageJ 1.41o (Wayne Rasband, National Institutes of Health; http://rsb.info.nih.gov/ij).

### Data analysis

The data regarding the immunophenotypic analysis were expressed descriptively as the mean values $\left(\overline{x}\right)$ and standard error of the means. The Wilcoxon rank-sum test was used to compare the values of the CG and TG for the hematological and CSF variables at the two time points (pre- and post-transplantation). To compare the values for the CSF and hematological variables at the two time points, the Wilcoxon signed rank test for paired samples was used. All analyses were conducted using SAS statistical software version 9.3 [44], with probability set at 5%.

## Abbreviations

MSCs: Mesenchymal stem cells
MMP: Matrix metalloproteinases
BM: Bone marrow
CSF: Cerebrospinal fluid
BM-MSCs: Bone marrow mesenchymal stem cells
CG: Control group
TG: Treated group
FBS: Fetal bovine serum
AS: Autologous serum
CNS: Central nervous system
PBS: Phosphate-buffered solution
